# Mental health in Ukraine in 2023

**DOI:** 10.1192/j.eurpsy.2024.12

**Published:** 2024-03-27

**Authors:** Dmytro Martsenkovskyi, Mark Shevlin, Menachem Ben-Ezra, Kristina Bondjers, Robert Fox, Thanos Karatzias, Inna Martsenkovska, Igor Martsenkovsky, Elisa Pfeiffer, Cedric Sachser, Frédérique Vallières, Philip Hyland

**Affiliations:** 1 Department of Psychiatry and Narcology, Bogomolets National Medical University, Kyiv, Ukraine; 2 SI Institute of Psychiatry, Forensic Psychiatric Examination and Drug Monitoring of Ministry of Health of Ukraine, Kyiv, Ukraine; 3 School of Psychology, Ulster University, Derry, Northern Ireland; 4 School of Social Work, Ariel University, Ariel, Israel; 5 Norwegian Centre for Violence and Traumatic Stress Studies, Oslo, Norway; 6 School of Business, National College of Ireland, Dublin, Ireland; 7 School of Health and Social Care, Edinburgh Napier University, Scotland; 8 Department of Child and Adolescent Psychiatry and Psychotherapy, Ulm University, Ulm, Germany; 9 Trinity Centre for Global Health, University of Dublin, Trinity College, Dublin, Ireland; 10 Department of Psychology, Maynooth University, Kildare, Ireland

**Keywords:** mental health, ukraine, comorbidity, prevalence, depression

## Abstract

**Background:**

Very little is known about the mental health of the adult population of Ukraine following Russia’s full-scale invasion in February 2022. In this study, we estimated the prevalence of seven mental health disorders, the proportion of adults screening positive for any disorder, and the sociodemographic factors associated with meeting requirements for each and any disorder.

**Methods:**

A non-probability quota sample (*N* = 2,050) of adults living in Ukraine in September 2023 was collected online. Participants completed self-report questionnaires of the seven mental health disorders. Logistic regression was used to determine the predictors of the different disorders.

**Results:**

Prevalence estimates ranged from 1.5% (cannabis use disorder) to 15.2% (generalized anxiety disorder), and 36.3% screened positive for any of the seven disorders. Females were significantly more likely than males (39.0% vs. 33.8%) to screen positive for any disorder. Disruption to life due to Russia’s 2014 invasion of Ukraine, greater financial worries, and having fewer positive childhood experiences were consistent risk factors for different mental health disorders and for any or multiple disorders.

**Conclusion:**

Our findings show that approximately one in three adults living in Ukraine report problems consistent with meeting diagnostic requirements for a mental health disorder 18 months after Russia’s full-scale invasion. Ukraine’s mental healthcare system has been severely compromised by the loss of infrastructure and human capital due to the war. These findings may help to identify those most vulnerable so that limited resources can be used most effectively.

## Background

The first epidemiological study of mental health disorders in Ukraine occurred in 2002 [1]. Face-to-face interviews were conducted with a probability-based sample of 4,725 adults, and the lifetime prevalence rates for nine disorders ranged from 0.3% (agoraphobia) to 14.6% (major depressive disorder [DD]). Overall, 31.6% met requirements for any disorder, with significantly higher rates in men. Men were nine times more likely to have an alcohol-related disorder, while women were about twice as likely to have a mood or anxiety disorder. Other demographic variables associated with having a mental health disorder included older age (mood disorders); living in the eastern and Kyiv regions versus the western region (mood, anxiety, and intermittent explosive disorders); being divorced or separated (all disorders); having less than a university education (mood, alcohol, and intermittent explosive disorders); not being employed (all disorders); and poorer financial status (mood disorders). More recent figures come from the 2017 Global Burden of Diseases study with rates of 3.4% for major DD, 6.0% for alcohol use disorder (AUD), and 0.7% for other drug use disorders being estimated [2].

The profound challenges that Ukraine has faced during the last decade may have affected the mental health of the population. In 2014, following the Maidan Revolution, Russia annexed Crimea and commenced a land war in the east of Ukraine giving rise to two million internally displaced people. Research with a representative sample of these people found that 27.4% met diagnostic requirements for post-traumatic stress disorder (PTSD), 20.6% for major DD, and 16.1% for generalized anxiety disorder (GAD), while 14.3% men and 1.7% of women screened positive for AUD [3]. In early 2020, Ukraine faced the COVID-19 pandemic and suffered its attendant social, economic, and public health effects. Following 2 years of social and economic disruption due to the pandemic, and 8 years of Russian aggression, Russia launched a full-scale invasion in February 2022. Research soon after this invasion revealed high levels of exposure to war-related stressors and post-traumatic stress symptoms in adults [4] and children [5], as well as sharp increases in anxiety and depression symptoms in adults [6] and children [7]. Although human beings are remarkably resilient to large-scale disasters [8], it is possible that these events have negatively affected the mental health of the population.

The primary objective of this study was to estimate the prevalence of mental distress in the general adult population of Ukraine 18 months after Russia’s full-scale invasion. The first objective was to determine a) the proportion of people meeting diagnostic requirements for PTSD, complex PTSD (CPTSD), prolonged grief disorder (PGD), DD, GAD, AUD, and/or cannabis use disorder (CUD), b) sex differences across all disorders and for any disorder, c) regional differences in the prevalence of disorders, and d) patterns of association between the disorders. The second objective was to identity the sociodemographic variables associated with meeting diagnostic requirements for each disorder. The third objective was to identify the sociodemographic variables associated with meeting diagnostic requirements for any disorder, and for multiple disorders.

## Methods

### Participants and procedures

This study is based on data collected as part of the “The Mental Health of Parents and Children in Ukraine Study: 2023 Follow-up”, which aims to record the social and mental health effects of Russia’s war on Ukraine [9]. An entirely new sample (*N* = 2,050) was recruited for the 2023 follow-up study. Data were collected by the survey research company TGM Research, which has access to nationally representative panels of nearly two million survey participants in over 130 countries, including Ukraine. Potential participants were contacted by TGM Research via email or in-app notification and were provided with a brief description of the study. Interested participants followed a link to a secure website operated by TGM Research, where they were provided with full details about the nature of the study. Participants providing informed consent completed the survey online. Ethical approval was provided by the SI Institute of Psychiatry, Forensic Psychiatric Examination and Drug Monitoring of Ministry of Health of Ukraine, Kyiv, Ukraine.

Data collection took place from September 7 to 18, 2023. Participants were selected using non-probability quota sampling methods to construct a sample intended to be representative of the Ukrainian population with respect to sex, age, and living location in Ukraine. It is, however, impossible to determine how representative our sample is of the current population of Ukraine. The last census in Ukraine took place in 2001 [10], and recent events have led to mass internal displacement and emigration, as well as many areas remaining under Russian occupation.

Details on statistical power and comparisons of the sample quotas to census-derived population figures are presented in the Supplementary Materials. We were unable to meet our quota of participants aged 60 years and older, and it was not possible to collect responses from Crimea. On all other quota variables, our sample is a reasonably close approximation of the population parameters. Attention checks were used in the survey (participants were asked, e.g., to select the third option from a list of 10 choices) and geolocation data (longitude and latitude of the terminal being used to complete the survey) were collected to ensure accurate regional distributions. Demographic information is presented in Table 1.

**Table 1.** Sociodemographic information for the sample (*N* = 2,050)

	%	N
Sex		
Males	51.7	1059
Females	48.3	991
Age		
18–29	20.8	426
30–39	25.2	517
40–49	23.2	475
50–59	19.0	390
60 and older	11.8	242
Born in Ukraine (or USSR)	92	1885
Current living location in Ukraine		
Western region	24.3	498
Northern region	22.0	450
Central region	13.5	277
Eastern region	15.6	319
Southern region	24.7	506
Life disrupted by 2014 invasion	42.2	866
Forced displacement by 2022 invasion	27.7	568
Living area		
Urban area	81.1	1662
Rural area	18.9	388
Type of living accommodation		
Apartment	64.1	1314
House	32.9	674
Hostel, hotel, collective accommodation center, or other form of emergency accommodation	3.0	62
Marital status		
Single	22.6	463
In a relationship but not living with a partner	4.1	84
In a relationship and living with a partner	10.7	219
Married	50.1	1027
Separated but still legally married	0.7	14
Divorced	8.3	171
Widowed	3.5	72
Have children	69.5	1425
Highest education level		
Completed mandatory schooling	2.8	58
Completed general/secondary schooling	10.9	223
Completed vocational school	28.4	582
Completed university	57.9	1187
Employment status		
Full–time employed	47.3	969
Part–time employed	19.6	401
Temporarily unemployed due to the war	7.8	159
Long–term unemployed	9.1	187
Student	3.5	72
Retired	10.3	211
Disabled and unable to work	2.5	51
Financial worry		
Not worried at all	3.9	79
A little worried	19.3	395
Moderately worried	30.0	614
Very worried	32.7	671
Extremely worried	14.2	291

### Measures

All data were collected via self-reports and completed in Ukrainian. The diagnostic criteria used to estimate the prevalence of each disorder are described in the Supplementary Materials.

#### ICD-11 DD and GAD

The International Depression Questionnaire (IDQ) [11] and the International Anxiety Questionnaire (IAQ) [11] ask participants to indicate how frequently they have experienced the nine DD symptoms over the last 2 weeks, and the eight GAD symptoms over the last several months, on a 5-point Likert scale (0 = never, 4 = every day). Participants also indicate if these symptoms have caused impairments in their daily life using a “yes” or “no” response format. The psychometric properties of the IDQ and IAQ are well supported [12], and internal reliability of the scale scores in this sample was excellent (IDQ, *ω* = .94; IAQ *ω* = .93).

#### ICD-11 PTSD and CPTSD

Participants were screened for trauma exposure using the International Trauma Exposure Measure [13], and those reporting a traumatic event (90.0%) completed the International Trauma Questionnaire (ITQ) [14]. The ITQ includes 12 items measuring PTSD and CPTSD symptoms, and participants report how bothered they have been by these symptoms over the past month on a 5-point Likert scale (0 = not at all, 4 = extremely). Six items measure functional impairment related to these symptoms. There is strong empirical support for the reliability and validity of the ITQ scores [15], including the Ukrainian translation [16], and internal reliability of the scale scores in this sample was good (*ω* = .89).

#### ICD-11 PGD

The International Grief Questionnaire (IGQ) [17] was completed by participants reporting a lifetime bereavement (87.7%). Participants report how bothered they have been by the five PGD symptoms over the past week on a 5-point Likert scale (0 = not at all, 4 = extremely), and if these symptoms caused functional impairment. The internal reliability of the scale scores in this sample was good (*ω* = .86).

#### Alcohol use disorder

The three-item AUD Identification Test-Concise (AUDIT-C) [18] is a shortened version of the World Health Organization’s AUDIT scale assessing hazardous drinking. Participants were asked about their drinking behavior over last 6 months, and responses are recorded on a 0- to 4-point Likert scale with separate options for each question. Total scores range from 0 to 12, and scores ≥5 indicate a probable drinking problem [19]. The psychometric properties of the AUDIT-C are strong [20] and the internal reliability of the scores in this sample was acceptable (*ω* = .67).

#### Cannabis use disorder

The short form of the CUD identification test-revised [21] includes four items. The first item screens for cannabis use in the past 6 months (“Yes” or “No”), and three questions assess hazardous use. These items are answered on a 5-point Likert scale (0 = never, 4 = daily or almost daily) and scores range from 0 to 12. Scores ≥2 are used to identify cases of CUD [21]. The internal reliability of the scale scores in this sample was excellent (*ω* = .90).

#### Positive memories of home and family life

Participants also completed the short form of the Memories of Home and Family Scale (MHFS-SF) [22], which includes 12 items measuring positive aspects of family life during the first 16 years of life. Participants report how frequently they experienced different events on a 5-point Likert scale (0 = never, 4 = always), with higher scores reflecting more positive memories of home and family life. Internal reliability of the MHFS-SF scores in this sample was excellent (*ω* = .97).

### Data analysis

Proportions meeting diagnostic requirements for each, and any, disorder were calculated, and chi-square (*χ*^2^) tests were used to determine sex and regional differences. Tetrachoric correlations were used to model the associations between the disorders. Binary logistic regression analysis was used to estimate the unique associations between the different predictor variables and meeting diagnostic requirements for each disorder (see Table 3 for the predictor variables). Multinomial logistic regression analysis was used to determine the association between the same predictor variables and meeting requirements for one, and two or more disorders, versus no disorder.

## Results

**Figure 1.**
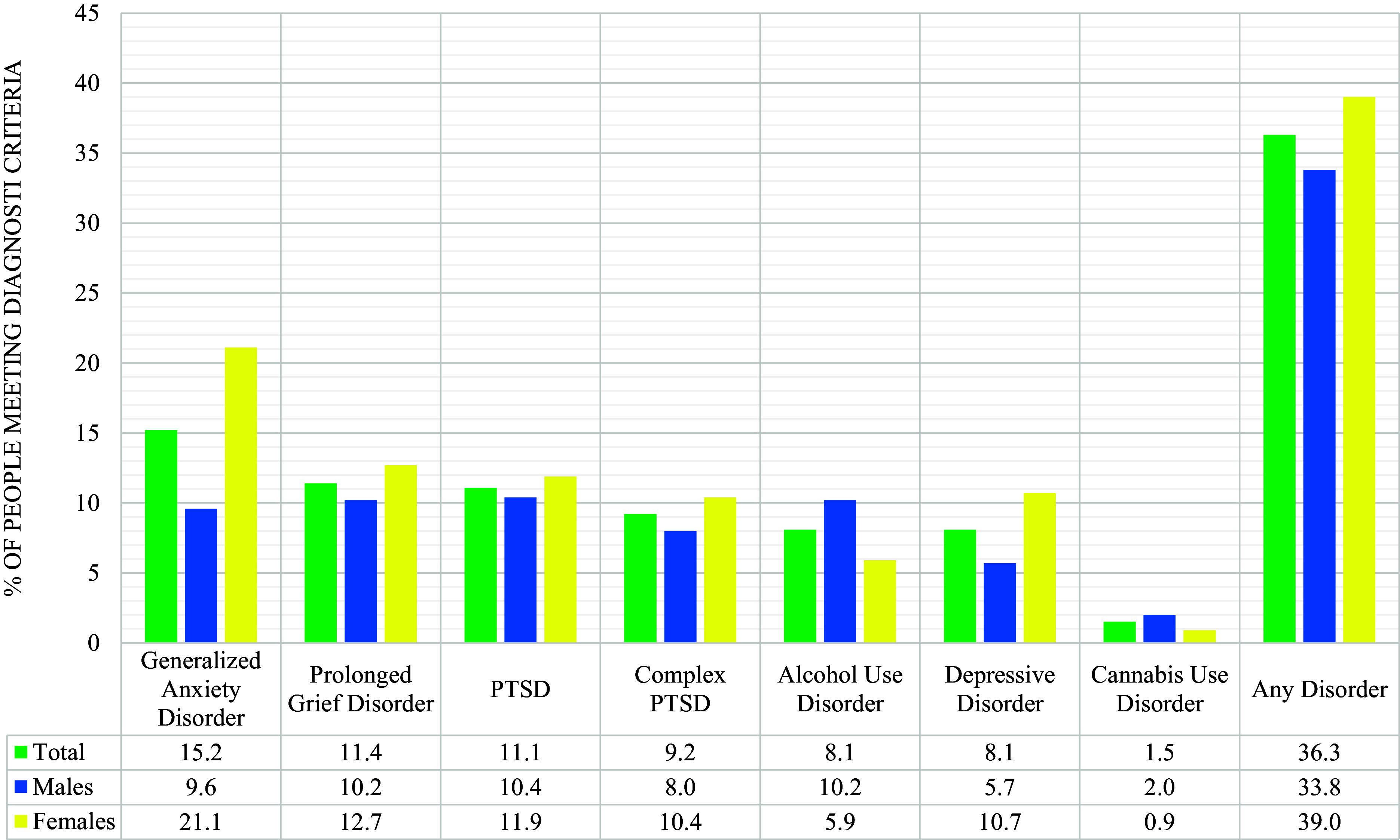
Percentage meeting criteria for each mental health disorder and of any mental health disorder for males, females, and entire sample.

Prevalence estimates are presented in Figure 1, ranging from 1.5% (CUD) to 15.2% (GAD). Overall, 36.3% met requirements for any disorder, with 20.1% meeting requirements for one disorder, and 16.2% for two or more disorders.

Females were significantly more likely to meet requirements for GAD (*χ* ^2^ (1) = 52.23, *p* < .001, OR = 2.51 [95% CI = 1.94, 3.24]) and DD (*χ* ^2^ (1) = 17.41, *p* < .001, OR = 1.99 [95% CI = 1.44, 2.77]), whereas males were significantly more likely to screen positive for AUD (*χ* ^2^ (1) = 12.99, *p* < .001, OR = 1.83 [95% CI = 1.31, 2.55]) and CUD (*χ* ^2^ (1) = 4.10, *p* = .043, OR = 2.21 [95% CI = 1.01, 4.84]). Females were significantly more likely to screen positive for any disorder (39.0% vs. 33.8%, *χ* ^2^ (1) = 5.86, *p* = .015, OR = 1.25 [95% CI = 1.04, 1.50]), and for two or more disorders (19.8% vs. 12.8%, *χ* ^2^ (2) = 18.15, *p* < .001, *φ* = .09).

Table 2 presents the proportions meeting criteria for disorders in each region of Ukraine. Significant differences were evident for GAD (*χ* ^2^ (4) = 9.60, *p* = .048, *φ* = .07), AUD (*χ* ^2^ (4) = 10.59, *p* = .032, *φ* = .07), and any disorder (*χ* ^2^ (4) = 12.56, *p* = .014, *φ* = .08). Rates of GAD were significantly higher than expected in the east and lower in the west. Rates of AUD were significantly higher in the north and lower in the south. Across all disorders, rates were significantly higher than expected in the east and lower than expected in the west.

**Table 2.** Percentages across different regions of Ukraine

	Overall	Western	Northern	Central	Eastern	Southern	***χ*** ^***2***^ **(p)**
PTSD	11.1	8.2	11.3	13.7	11.0	12.5	7.03 (.135)
CPTSD	9.2	7.0	10.2	8.3	12.9	8.5	9.06 (.060)
PGD	11.4	11.6	10.7	11.9	10.3	12.3	1.06 (.901)
GAD	15.2	**12.0** ^a^	14.2	17.3	**19.4** ^b^	15.2	**9.60 (.048)**
DD	8.1	7.4	7.6	8.7	10.3	7.5	3.00 (.559)
AUD	8.1	8.8	**10.7** ^b^	5.8	9.1	**5.7** ^a^	**10.59 (.032)**
CUD	1.5	1.2	2.0	0.7	1.9	1.4	2.59 (.628)
Any disorder	36.3	**30.5** ^a^	38.9	35.7	41.7	**36.6** ^b^	**12.56 (.014)**

Abbreviations: DD, depressive disorder; GAD, generalized anxiety disorder; PTSD, post-traumatic stress disorder; CPTSD, complex PTSD; PGD, prolonged grief disorder; AUD, alcohol use disorder; CUD, cannabis use disorder; *χ^2^* (p), chi-square test and associated level of statistical significance (all tests have 4 degrees of freedom).

*Note: Statistically significant (p < .05) effects are in bold.*

a Percentage of people in this cell is significantly smaller than the overall figure.

b Percentage of people in this cell is significantly larger than the overall figure.

**Figure 2.**
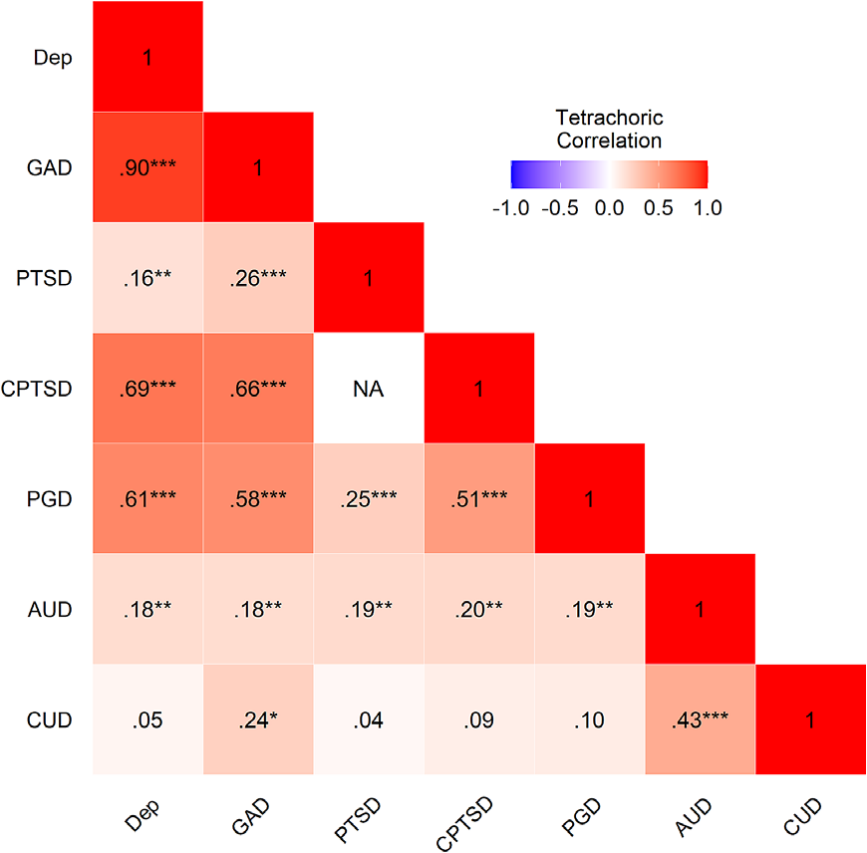
Tetrachoric correlations among all mental health disorders. Note: No correlation was provided for PTSD and CPTSD as these disorders are independent by the nature of their diagnostic rules; DD, depressive disorder; GAD, generalized anxiety disorder; PTSD, ICD-11 post-traumatic stress disorder; CPTSD, ICD-11 complex PTSD; PGD, prolonged grief disorder; AUD, alcohol use disorder; CUD, cannabis use disorder.

Figure 2 presents the tetrachoric correlations between the seven disorders. All correlations were positive and 16 of 20 were statistically significant. The strongest correlation was between DD and GAD (*r* = .90). CUD had the weakest pattern of association with other disorders, only correlating significantly with AUD (*r* = .43) and GAD (*r* = .24).

The adjusted associations between the predictor variables and meeting requirements for each disorder are presented in Table 3, and all logistic regression models were statistically significant (*p* < .001).

**Table 3.** Odds ratios for risk factors associated with meeting diagnostic requirements for each disorder

	PTSD	CPTSD	PGD	GAD	DD	AUD	CUD
Female sex	1.17	1.06	1.27	**2.42**	**1.81**	**0.55**	**0.30**
18–29 years old^a^	0.99	2.36	0.62	**2.42**	1.32	1.18	7.34
30–39 years old^a^	1.13	1.62	0.89	**2.13**	1.40	1.30	3.51
40–49 years old^a^	1.73	1.28	0.81	1.39	0.67	1.24	3.29
50–59 years old^a^	1.71	1.20	0.59	1.47	0.89	0.61	2.11
University education	1.18	**1.46**	0.77	1.07	1.15	0.84	**0.22**
Part–time employed^b^	1.06	0.81	0.83	0.88	0.74	0.64	1.06
Unemployed^b^	0.83	1.03	0.71	0.89	1.03	0.84	1.62
Student^b^	1.34	0.81	0.85	1.33	1.79	**0.21**	0.92
Retired^b^	0.83	0.73	0.67	1.35	0.93	**0.39**	2.56
Financial worries	1.08	**1.71**	**1.26**	**1.69**	**1.98**	0.98	1.00
Single^c^	1.00	1.20	0.93	1.06	1.42	0.84	0.68
Divorced, separated, or widowed^c^	1.09	**1.76**	**1.92**	1.05	1.01	1.01	1.30
Number of children	1.14	0.90	0.98	1.07	1.07	0.87	0.91
North Ukraine^d^	1.32	0.95	0.79	1.01	0.80	1.12	1.30
Central Ukraine^d^	**1.68**	0.98	0.85	1.27	0.90	0.63	0.54
East Ukraine^d^	1.20	1.09	0.69	1.31	1.01	0.93	1.10
South Ukraine^d^	1.51	0.96	0.95	1.21	0.88	**0.60**	1.00
Urban living	1.02	1.31	0.99	1.16	1.31	1.27	1.21
2014 life disruption	**1.83**	**1.75**	**2.52**	**2.26**	**2.06**	**1.68**	1.00
2022 displacement	0.91	**1.66**	0.82	0.99	0.99	0.80	**2.45**
Positive memories of home and family life	0.99	**0.97**	**0.98**	**0.97**	**0.97**	**0.98**	**0.95**
Overall model *χ* ^2^ (*df* = 22)	**51.04**	**150.13**	**97.28**	**219.07**	**159.70**	**75.63**	**55.50**

Abbreviations: PTSD, post-traumatic stress disorder; CPTSD, complex PTSD; PGD, prolonged grief disorder; GAD, generalized anxiety disorder; DD, depressive disorder; AUD, alcohol use disorder; CUD, cannabis use disorder; *χ^2^* (p), chi-square test and associated level of statistical significance (all tests have 4 degrees of freedom).

*Note:* statistically significant effects (*p* < .05) are in bold.

a Comparison category is 60 years and older.

b Comparison category is full-time employed.

c Comparison category is married or in a committed relationship.

d Comparison category is living in Western Ukraine.

Regarding the stress-related disorders, meeting criteria for PTSD was associated with living in central Ukraine and being affected by the 2014 Russian invasion. Meeting criteria for CPTSD was associated with having a university education, greater financial worries, being divorced, separated, or widowed, being affected by the 2014 Russian invasion, being displaced by the 2022 Russian invasion, and having fewer positive memories of home and family life. Meeting criteria for PGD was associated with greater financial worries, being divorced, separated, or widowed, being affected by the 2014 Russian invasion, and fewer positive memories of home and family life.

Regarding the anxiety and mood disorders, meeting requirements for GAD was associated with being female, being aged 18–29 and 30–39 years, greater financial worries, being affected by the 2014 Russian invasion, and fewer positive memories of home and family life. Meeting requirements for DD was associated with being female, greater financial worries, being affected by the 2014 Russian invasion, and fewer positive memories of home and family life.

Finally, regarding the substance misuse disorders, screening positive for AUD was associated with being male, being affected by the 2014 Russian invasion, and fewer positive memories of home and family life. Additionally, screening positive for AUD was less likely among students, those retired, and those living in the south of Ukraine. Screening positive for CUD was associated with being male, not having a university education, being displaced by the 2022 Russian invasion, and fewer positive memories of home and family life.

The multinomial logistic regression model predicting those meeting diagnostic requirements for one and two or more disorders versus zero disorders was statistically significant (*χ* ^2^ (44) = 285.39, *p* < .001). Adjusted associations are reported in Table 4.

**Table 4.** Odds ratios associated with meeting diagnostic requirements for different numbers of disorders

	One disorder	Two or more disorders
Female sex	0.93	**1.50**
18–29 years old^a^	0.93	**2.21**
30–39 years old^a^	0.88	1.88
40–49 years old^a^	0.99	1.53
50–59 years old^a^	0.94	1.17
University education	1.24	1.06
Part–time employed^b^	1.02	0.82
Unemployed^b^	0.74	0.88
Student^b^	0.96	1.04
Retired^b^	0.72	0.91
Financial worries	1.06	**1.66**
Single^c^	0.87	1.20
Divorced, separated, or widowed^c^	1.23	**1.64**
Number of children	**1.18**	1.02
North Ukraine^d^	**1.52**	0.95
Central Ukraine^d^	1.19	1.05
East Ukraine^d^	**1.64**	0.95
South Ukraine^d^	**1.40**	1.03
Urban living	1.13	1.28
2014 life disruption	**1.54**	**2.63**
2022 displacement	0.88	1.78
Positive memories of home and family life	**0.97**	**0.97**

*Note.* All effects are adjusted odds ratios derived from binary logistic regression analyses; statistically significant effects (*p* < .05) are in bold.

a Comparison category is 60 years and older.

b Comparison category is full-time employed.

c Comparison category is married or in a committed relationship

d Comparison category is living in Western Ukraine.

Meeting criteria for a single disorder was associated with having more children, living in northern, eastern, and southern Ukraine, being affected by the 2014 Russian invasion, and having lower levels of positive memories of home and family life. Meeting criteria for two or more disorders was associated with being female, being aged 18–29 years, having more financial worries, being divorced, separated, or widowed, being affected by the 2014 Russian invasion, and having fewer positive memories of home and family life.

## Discussion

This study was conducted to estimate the level of psychological distress in the Ukrainian population 18 months after Russia’s full-scale invasion. Approximately one in three adults reported problems consistent with meeting diagnostic requirements for one of seven disorders. The most common problem was GAD (15.2%), while CUD was the least common problem (1.5%). Between 8% and 12% of people screened positive for the other stress, mood, and substance misuse disorders. Comorbidity between disorders was common, and half of those meeting criteria for one disorder met criteria for at least one other disorder. Methodological differences mean it is impossible to compare current findings to prior studies from Ukraine [1], but both studies found that about one-third of adults were experiencing clinically relevant mental health problems. This suggests that the overall prevalence of mental health disorders in Ukraine is not markedly different from what is seen in other Western and European nations [23,24]. These findings may be taken as a sign of the remarkable resilience of the Ukrainian population, given the immense challenges they have faced in recent years.

We observed the typical pattern of sex differences in disorders that has been found in Ukraine [1,2], and internationally [25]. Women were 2–2.5 times more likely to meet diagnostic requirements for GAD and DD while men were about two times more likely to screen positive for AUD and CUD. These differences remained when controlling for all other sociodemographic variables, and in the case of CUD, the effect increased in magnitude such that men were nearly 3.5 times more likely to screen positive. Notably, we found that women were more likely than men to screen positive for any disorder, and for two or more disorders. Adjusting for all other sociodemographic variables, the effect of screening positive for two or more disorders remained significant. Among Ukrainian adults, it was previously found that men were significantly more likely than women to have a disorder, but this was likely due to the stark differences in alcohol misuse disorders [1]. Current findings suggest that women differ from men not only in the type of disorders they are more likely to report, but also in their risk for disorder comorbidity.

There were some differences in disorder prevalence across geographical regions of Ukraine, with people living in the east most likely to screen positive for a disorder, and people in the west the least likely. This is perhaps not surprising given that the east of Ukraine has been under Russian attack since 2014 and continues to be on the front line of the ongoing war. Adjusting for all other sociodemographic variables, we found that people living in the east, south, and north of Ukraine were more likely to screen positive for a mental health disorder than people living in the west. These findings should be interpreted cautiously. Although we were able to recruit people living in the eastern and southern parts of Ukraine, people most severely affected by the war in these areas may not have been contactable due to the requirement for internet access. Furthermore, millions of people have left the most severely affected regions of Ukraine (east and south) since 2014 for refuge in other, safer parts of the country meaning the composition of different regions is changing rapidly over short time scales. It is difficult therefore to offer a simple explanation for why some disorders may be higher in one region than another, but we hope these findings have some value in indicating where mental health resources may be most needed.

Across our analyses, several other sociodemographic variables emerged as robust correlates of different disorders. Aligning with prior research [3], people who indicated that their lives were disrupted by Russia’s 2014 invasion (meaning they were forced to move, lost property, became disabled, or were bereaved) were upward of 2.5 times more likely to screen positive for each disorder (other than CUD) and to screen positive for two or more disorders. Nearly half the sample (47%) reported feeling “extremely” or “very” worried about their financial security, and financial worries were associated with almost all disorders. Financial insecurity is increasingly recognized as a major risk factor for mental disorder [26,27]. Solutions to mental health problems are often thought to lie in psychotherapeutic or pharmacological interventions, but if a major cause of distress is financial insecurity, a solution might also be found in political and economic interventions. Ensuring the continued financial security of the people of Ukraine is therefore a key humanitarian task for the international community.

Positive memories of home and family life were associated with a lower likelihood of meeting criteria for every disorder (except PTSD) and, notably, the effect was especially strong for CUD. Positive childhood experiences are known to play a key role in protecting people from developing mental health disorders later in life [28], even in those with histories of childhood trauma [29]. In the current sample, scores on this variable were highly negatively skewed, meaning that most people reported very positive memories of their home and family life. This is encouraging and may be one factor in explaining the resilience of the population. Moreover, this variable may offer a useful means of identifying those most at risk for a mental health disorder, especially CUD.

We found that younger adults (i.e., those aged 18–40) were more than twice as likely to meet requirements for GAD than those over 60 years, and the youngest cohort (i.e., 18–29 years old) were more than twice as likely to screen positive for two or more disorders. Previous work in Ukraine indicated that older adults were somewhat more likely to have a mental health disorder [1]; a finding inconsistent with the wider literature [30]. It is possible these discrepant findings can be explained by cohort effects, but regardless of the reason, current findings suggest that younger adults in Ukraine are most likely to report mental health problems. Those with a university education were nearly five times *less likely* than those without a university education to screen positive for CUD, and full-time students were *less likely* to screen positive for AUD. This suggests that alcohol and substance misuse is strongly related to lower educational attainment in Ukraine. Finally, individuals who were divorced, separated, or widowed were nearly twice as likely to meet criteria for CPTSD and PGD than those who were married or in a committed relationship, and were 64% more likely to meet requirements for two or more disorders. It is noteworthy that single people were no more likely to have a disorder, meaning that being in a relationship is not necessarily protective; rather, the dissolution of a relationship seems to be key [1].

This study contains several limitations. Non-probability-based sampling methods were used, and we were unable to recruit our desired quota of older adults or people from some areas of Ukraine under Russian occupation (e.g., Crimea). While we endeavored to construct the sample in a way that was representative of the population across several demographic variables, it is unlikely that our sample completely represents the population. Second, we were only able to measure seven disorders, and these do not represent the entire spectrum of mental health problems. Third, our prevalence estimates were derived from self-report data, which can yield higher estimates relative to clinical assessment, but we used diagnostic algorithm scoring methods, where possible (i.e., PTSD, CPTSD, PGD, GAD, DD), and employed conservative cut-off scores where diagnostic algorithms were not available (i.e., AUD, CUD) to minimize possible type 1 errors. Finally, the cross-sectional design means no causal interpretations can be made regarding the relationships between the sociodemographic and mental health variables.

This study provides important information about the state of mental health in the adult population of Ukraine in 2023. It appears that about one-third of adults have clinically relevant problems related to anxiety, traumatic stress, bereavement, low mood, and substance misuse. Key risk factors for these mental health problems include younger age, lower education attainment, greater financial worries, no longer being in a committed relationship, having fewer positive childhood experiences within one’s family context, and experiencing serious disruption to one’s life due to Russian aggression. Meeting this level of need is going to be challenging because the Ukrainian mental healthcare system was considered to be severely under-resourced before the onset of the full-scale invasion, and has experienced serious infrastructure and personnel damage since then [31]. It is our hope that these findings help in some way to understand the level of need, and who is in need, so that limited resources can be used most effectively.

## Supporting information

Martsenkovskyi et al. supplementary material Martsenkovskyi et al. supplementary material

## Data Availability

All data are available upon request from the corresponding author.

## Supplementary material

The supplementary material for this article can be found at http://doi.org/10.1192/j.eurpsy.2024.12.
