# Supplementary material for: Mental health in Ukraine in 2023
Source: Eur Psychiatry. 2024 Mar 27;67(1):e27. doi: 10.1192/j.eurpsy.2024.12 (PMC10988158; doi:10.1192/j.eurpsy.2024.12)
Supplement: Martsenkovskyi et al. supplementary material [file S0924933824000129sup001.docx]

1. **Statistical power**

An *a priori* power analysis was conducted to estimate the sample size needed to detect a disorder with a 5% prevalence rate, in a population of 37 million people, with a 1% margin of error, and a confidence level of 95%. This resulted in a required sample size of 1,825 people. In consultation with the survey company employed to collect the data, we agreed on a target sample sized of 2,000 people. In total, we collected responses from 2,050 people.

1. **Census data and sample quotas**

The table below presents the estimated population parameters for sex, age, and regional distribution alongside the composition of our obtained sample. For note, the last census in Ukraine was conducted in 2001, although estimates are available from 2022.

**Supplementary Table. Comparison between census data and sample composition across the three quota variables for sample selection.**

|  | Census figures | Sample composition |
| --- | --- | --- |
| ***Sex*** |  |  |
| Male | 50.0% | 51.7% |
| Female | 50.0% | 48.3% |
| ***Age*** |  |  |
| 18-29 | 16.0% | 20.8% |
| 30-39 | 21.0% | 25.2% |
| 40-49 | 19.0% | 23.2% |
| 50-59 | 17.0% | 19.0% |
| 60 and older | 26.0% | 11$.$8% |
| ***Region*** |  |  |
| Western Ukraine (Lvivska oblast, Ivano-Frankivska oblast, Zakarpatska oblast, Rivnenska oblast, Ternopilska oblast, Volynska oblast, Chernivetska oblast, Khmelnytska oblast) | 26.0% | 24.3% |
| North Ukraine (Zhytomyrska oblast, Kyivska oblast, Chernihivska oblast, Sumska oblast) | 19.0% | 22.0% |
| Central Ukraine (Vinnytska oblast, Kirovohradska oblast, Poltavska oblast, Cherkaska oblast) | 14.0% | 13.5% |
| Eastern Ukraine (Donetska oblast, Kharkivska oblast, Luhanska oblast) | 18.0% | 15.6% |
| South Ukraine (Zaporizka oblast, Dnipropetrovska oblast, Khersonska oblast, Odeska oblast, Mykolaivska oblast) | 23.0% | 24.7% |

1. **Diagnostic criteria for the different disorders**

**ICD-11 Depressive Disorder**: We assessed depression using the International Depression Questionnaire which measures all diagnostic criteria for Single Episode Depressive Disorder in the 11^th^ version of the International Classification of Diseases (ICD-11) (code 6A70). Participants indicated how frequently they have experienced each of the nine symptoms over the last two weeks using a five-point Likert scale (0 = Never, 1 = Only a few days, 2 = Half the days, 3 = Most days, and 4 = Every day). They are also indicated if these symptoms caused impairment in their daily life using a ‘Yes’ or ‘No’ response format. The diagnostic requirement for Depressive Disorder in the ICD-11 imply that a symptom is ‘present’ based on responses of > 3 on the Likert scale. Meeting diagnostic requirements requires the presence of five or more symptoms, where at least one of the two ‘essential’ symptoms (i.e., feeling down or depressed for most of the day and diminished interest or pleasure from normal activities for most of the day) is present, plus functional impairment.

**ICD-11 Generalized Anxiety Disorder**: We assessed anxiety using the International Anxiety Questionnaire which measures all diagnostic criteria for Generalized Anxiety Disorder in ICD-11 (code 6B00). Participants are asked to indicate how frequently they have experienced each symptom over the last several months using a five-point Likert scale (0 = Never, 1 = Only a few days, 2 = Half the days, 3 = Most days, and 4 = Every day). They are also asked to indicate if these symptoms have caused impairment in their daily life using a ‘Yes’ or ‘No’ response format. As per the ICD-11 guidelines, a symptom is ‘present’ based on responses of > 3 on the Likert scale. Meeting diagnostic requirements involves the presence of four or more symptoms, where at least one of the two ‘essential’ symptoms (i.e., feeling nervous or anxious, and worrying a lot about different things) is endorsed, plus functional impairment.

**ICD-11 Posttraumatic Stress Disorder and Complex Posttraumatic Stress Disorder**: We assessed these disorders using the International Trauma Questionnaire which captures all diagnostic criteria for PTSD (6B40) and Complex PTSD (6B41) in ICD-11. Diagnostic requirements for ICD-11 PTSD are met if there is evidence of (1) exposure to a traumatic event (this was assessed using the International Trauma Exposure Measure), (2) the traumatic exposure occurred more than one month ago, (3) the presence of least one symptom from the three PTSD symptom clusters of re-experiencing in the here and now, avoidance, and sense of current threat, and (4) the presence of functional impairment associated with these symptoms. Diagnostic requirements for ICD-11 CPTSD are met if all PTSD criteria are satisfied *and* at least one symptom from the three ‘Disturbance in Self-Organisation’ symptom clusters of affective dysregulation, negative self-concept, and disturbed relationships are present, and there is evidence of functional impairment associated with these symptoms. Participants report how bothered they have been by the symptoms over the past week using a five-point Likert scale (0 = Not at all, 1 = A little bit, 2 = Moderately, 3 = Quite a bit, and 4 = Extremely), and a symptom is deemed to be present based on responses > 2 on the Likert scale. Separate sets of three items are used to measure functional impairment associated with the PTSD symptoms, and the Disturbance in Self-Organization symptoms. The ICD-11 diagnostic rules permit a diagnosis of PTSD or CPTSD, but not both. Thus, if a person meets criteria for CPTSD, they do not also receive a PTSD diagnosis.

**ICD-11 Prolonged Grief Disorder**: We assessed prolonged grief disorder using the International Grief Questionnaire which capturing all diagnostic requirements for ICD-11 PGD (code 6B42). Diagnostic requirements for ICD-11 PGD are met if participants (1) are bereaved, (2) the bereavement occurred more than six months ago, (3) have at least one of the two ‘essential’ grief symptoms (i.e., yearning for the deceased almost every day or thinking too much about the deceased almost every day), (4) have at least one of three ‘accessory’ symptoms (i.e., feeling guilty or angry, having trouble accepting the death, and feeling sad or emotionally numb), (5) report functional impairment associated with these symptoms, and (6) indicate that their grief response has lasted for longer than would be expected based on their social, cultural, or religious context. Participants indicated how bothered they have been the five PGD symptoms over the past week using a five-point Likert scale (0 = Not at all, 1 = A little bit, 2 = Moderately, 3 = Quite a bit, and 4 = Extremely), and a symptom is deemed to be present based on responses > 2 on the Likert scale. Functional impairment related to the symptoms is assessed using a ‘Yes’ or ‘No’ response format.

**Alcohol Use Disorder:** We used the three-item Alcohol Use Disorders Identification Test-Concise (AUDIT-C) which asks participants about their drinking behaviour over last six months (i.e., ‘How often do you have a drink containing alcohol?’, ‘How many drinks containing alcohol do you have on a typical day when you are drinking?’, and ‘How often do you have six or more drinks on one occasion?’). Responses are recorded on a 0-4 Likert scale with separate response options for each question, and total scores range from 0-12. The United Kingdom government guidelines indicate that scores > 5 are indicative of hazardous drinking, and probable Alcohol Use Disorder status.

**Cannabis Use Disorder**: We used the short form of the Cannabis Use Disorder Identification Test-Revised (CUDIT-R) which contains four items. The first item screens for cannabis use in the past six months on a ‘Yes’ or ‘No’ scale. Those indicating cannabis use in the last six months then answer three questions assessing hazardous use (‘How often during the past 6 months did you find that you were not able to stop using cannabis once you had started?’, ‘How often in the past 6 months have you devoted a great deal of your time to getting, using, or recovering from cannabis?’, and ‘How often in the past 6 months have you had a problem with your memory or concentration after using cannabis?’). These items are answered on a five-point Likert scale (0 = Never, 1 = Less than monthly, 2 = Monthly, 3 = Weekly, and 4 = Daily or almost daily) and scores range from 0-12. The scale developers provided evidence that scores > 2 have good sensitivity and specificity for identifying cases of Cannabis Use Disorder.
